# STrengthening the REporting of Genetic Association studies (STREGA) – an extension of the STROBE statement

**DOI:** 10.1111/j.1365-2362.2009.02125.x

**Published:** 2009-04

**Authors:** Julian Little, Julian PT Higgins, John PA Ioannidis, David Moher, France Gagnon, Erik von Elm, Muin J Khoury, Barbara Cohen, George Davey-Smith, Jeremy Grimshaw, Paul Scheet, Marta Gwinn, Robin E Williamson, Guang Yong Zou, Kim Hutchings, Candice Y Johnson, Valerie Tait, Miriam Wiens, Jean Golding, Cornelia van Duijn, John McLaughlin, Andrew Paterson, George Wells, Isabel Fortier, Matthew Freedman, Maja Zecevic, Richard King, Claire Infante-Rivard, Alex Stewart, Nick Birkett

**Affiliations:** * Canada Research Chair in Human Genome EpidemiologyOttawa, ON, Canada; † University of OttawaOttawa, ON, Canada; ‡ MRC Biostatistics UnitCambridge, UK; § University of Ioannina, Ioannina, Greece; and Tufts University School of MedicineBoston, MA, USA; ¶ University of TorontoToronto, ON, Canada; ** University of Bern, Bern, Switzerland; and University Medical CentreFreiburg, Germany; †† National Office of Public Health GenomicsAtlanta, GA, USA; ‡‡ Public Library of ScienceSan Francisco, CA, USA; §§ University of BristolBristol, UK; ¶¶ University of OttawaOttawa, ON, Canada; *** University of Texas, MD Anderson Cancer CenterHouston, TX, USA; ††† American Journal of Human GeneticsBoston, MA, USA; ‡‡‡ University of Western Ontario; and Robarts Research InstituteLondon, Ottawa, ON, Canada; §§§ Paediatric and Perinatal EpidemiologyBristol, UK; ¶¶¶ European Journal of EpidemiologyRotterdam, The Netherlands; **** Cancer Care Ontario; and Prosserman Centre for Health Research at the Samuel Lunenfeld Research InstituteToronto, ON, Canada; †††† Canada Research Chair in Genetics of Complex DiseasesToronto, ON, Canada; ‡‡‡‡ University of Ottawa Heart InstituteOttawa, ON, Canada; §§§§ McGill University and Genome Quebec Innovation CenterMontreal, QC, Canada; ¶¶¶¶ Dana-Farber Cancer InstituteBoston, MA, USA; ***** LancetNew York, New York, USA; ††††† Genetics in MedicineMinneapolis, MN, USA; ‡‡‡‡‡ McGill University, MontrealQC, Canada; §§§§§ University of Ottawa Heart InstituteOttawa, ON, Canada

**Keywords:** Epidemiology, gene-disease associations, gene-environment interaction, genetics, genome-wide association, meta-analysis, reporting recommendations, systematic review

## Abstract

Making sense of rapidly evolving evidence on genetic associations is crucial to making genuine advances in human genomics and the eventual integration of this information in the practice of medicine and public health. Assessment of the strengths and weaknesses of this evidence, and hence the ability to synthesize it, has been limited by inadequate reporting of results. The STrengthening the REporting of Genetic Association studies (STREGA) initiative builds on the STrengthening the Reporting of OBservational Studies in Epidemiology (STROBE) Statement and provides additions to 12 of the 22 items on the STROBE checklist. The additions concern population stratification, genotyping errors, modelling haplotype variation, Hardy–Weinberg equilibrium, replication, selection of participants, rationale for choice of genes and variants, treatment effects in studying quantitative traits, statistical methods, relatedness, reporting of descriptive and outcome data and the volume of data issues that are important to consider in genetic association studies. The STREGA recommendations do not prescribe or dictate how a genetic association study should be designed, but seek to enhance the transparency of its reporting, regardless of choices made during design, conduct or analysis.

The rapidly evolving evidence on genetic associations is crucial to integrating human genomics into the practice of medicine and public health [1,2]. Genetic factors are likely to affect the occurrence of numerous common diseases and therefore, identifying and characterizing the associated risk (or protection) will be important in improving the understanding of etiology and potentially for developing interventions based on genetic information. The number of publications on the associations between genes and diseases has increased tremendously; with more than 34 000 published articles, the annual number has more than doubled between 2001 and 2008 [3,4]. Articles on genetic associations have been published in about 1500 journals and in several languages.

Despite the many similarities between genetic association studies and ‘classical’ observational epidemiologic studies (that is, cross-sectional, case-control, and cohort) of lifestyle and environmental factors, genetic association studies present several specific challenges including an unprecedented volume of new data [5,6] and the likelihood of very small individual effects. Genes may operate in complex pathways with gene-environment and gene-gene interactions [7]. Moreover, the current evidence base on gene-disease associations is fraught with methodological problems [8–10]. Inadequate reporting of results, even from well-conducted studies, hampers assessment of a study’s strengths and weaknesses and hence the integration of evidence [11].

Although several commentaries on the conduct, appraisal and/or reporting of genetic association studies have so far been published [12–39], their recommendations differ. For example, some papers suggest that replication of findings should be part of the publication [12,13,16,17,23,26,34–36], whereas others consider this suggestion unnecessary or even unreasonable [21,40–44]. In many publications, the guidance has focused on genetic association studies of specific diseases [14,15,17,19,22,23,25,26,31–38] or the design and conduct of genetic association studies [13–15,17,19,20,22,23,25,30–32,35,36] rather than on the quality of the reporting.

Despite increasing recognition of these problems, the quality of reporting genetic association studies needs to be improved [45–49]. For example, an assessment of a random sample of 315 genetic association studies published from 2001 to 2003 found that most studies provided some qualitative descriptions of the study participants (for example, origin and enrolment criteria), but reporting of quantitative descriptors such as age and gender was variable [49]. In addition, completeness of reporting of methods that allow readers to assess potential biases (for example, number of exclusions or number of samples that could not be genotyped) varied [49]. Only some studies described methods to validate genotyping or mentioned whether research staff were blinded to outcome. The same problems persisted in a smaller sample of studies published in 2006 [49]. Lack of transparency and incomplete reporting have raised concerns in a range of health research fields [11,50–53] and poor reporting has been associated with biased estimates of effects in clinical intervention studies [54].

The main goal of this article is to propose and justify a set of guiding principles for reporting results of genetic association studies. The epidemiology community has recently developed the STrengthening the Reporting of OBservational Studies in Epidemiology (STROBE) Statement for cross-sectional, case-control and cohort studies [55,56]. Given the relevance of general epidemiological principles for genetic association studies, we propose recommendations in an extension of the STROBE Statement called the STrengthening the REporting of Genetic Association studies (STREGA) Statement. The recommendations of the STROBE Statement have a strong foundation because they are based on empirical evidence on the reporting of observational studies and they involved extensive consultations in the epidemiological research community [56]. We have sought to identify gaps and areas of controversy in the evidence regarding potential biases in genetic association studies. With the recommendations, we have indicated available empirical or theoretical work that has demonstrated or suggested that a methodological feature of a study can influence the direction or magnitude of the association observed. We acknowledge that for many items, no such evidence exists. The intended audience for the reporting guideline is broad and includes epidemiologists, geneticists, statisticians, clinician scientists and laboratory-based investigators who undertake genetic association studies. In addition, it includes ‘users’ of such studies who wish to understand the basic premise, design, and limitations of genetic association studies to interpret the results. The field of genetic associations is evolving very rapidly with the advent of genome-wide association investigations, high-throughput platforms assessing genetic variability beyond common single nucleotide polymorphisms (SNPs) (for example, copy number variants, rare variants), and eventually routine full sequencing of samples from large populations. Our recommendations are not intended to support or oppose the choice of any particular study design or method. Instead, they are intended to maximize the transparency, quality and completeness of reporting of what was done and found in a particular study.

## Methods

A multidisciplinary group developed the STREGA Statement by using literature review, workshop presentations and discussion and iterative electronic correspondence after the workshop. Thirty-three of 74 invitees participated in the STREGA workshop in Ottawa, Ontario, Canada, in June, 2006. Participants included epidemiologists, geneticists, statisticians, journal editors and graduate students.

Before the workshop, an electronic search was performed to identify existing reporting guidance for genetic association studies. Workshop participants were also asked to identify any additional guidance. They prepared brief presentations on existing reporting guidelines, empirical evidence on reporting of genetic association studies, the development of the STROBE Statement and several key areas for discussion that were identified on the basis of consultations before the workshop. These areas included the selection and participation of study participants, rationale for choice of genes and variants investigated, genotyping errors, methods for inferring haplotypes, population stratification, assessment of Hardy–Weinberg equilibrium (HWE), multiple testing, reporting of quantitative (continuous) outcomes, selectively reporting study results, joint effects and inference of causation in single studies. Additional resources to inform workshop participants were the HuGENet handbook [57,58], examples of data extraction forms from systematic reviews or meta-analyses, articles on guideline development [59,60] and the checklists developed for STROBE. To harmonize our recommendations for genetic association studies with those for observational epidemiologic studies, we communicated with the STROBE group during the development process and sought their comments on the STREGA draft documents. We also provided comments on the developing STROBE Statement and its associated explanation and elaboration document [56].

## Results

In Table 1, we present the STREGA recommendations, an extension to the STROBE checklist [55] for genetic association studies. The resulting STREGA checklist provides additions to 12 of the 22 items on the STROBE checklist. During the workshop and subsequent consultations, we identified five main areas of special interest that are specific to, or especially relevant in, genetic association studies: genotyping errors, population stratification, modelling haplotype variation, HWE and replication. We elaborate on each of these areas, starting each section with the corresponding STREGA recommendation, followed by a brief outline of the issue and an explanation for the recommendations. Complementary information, on these areas and the rationale for additional STREGA recommendations relating to selection of participants, choice of genes and variants selected, treatment effects in studying quantitative traits, statistical methods, relatedness, reporting of descriptive and outcome data, and issues of data volume, are presented in Table 2.

**Table 1:** STREGA reporting recommendations, extended from STROBE statement

Item	Item number	STROBE guideline	Extension for genetic association studies (STREGA)
Title and abstract	1	(a) Indicate the study’s design with a commonly used term in the title or the abstract	
		(b) Provide in the abstract an informative and balanced summary of what was done and what was found	
Introduction			
Background rationale	2	Explain the scientific background and rationale for the investigation being reported	
Objectives	3	State specific objectives, including any pre-specified hypotheses	State if the study is the first report of a genetic association, a replication effort or both
Methods			
Study design	4	Present key elements of study design early in the paper	
Setting	5	Describe the setting, locations and relevant dates, including periods of recruitment, exposure, follow-up and data collection	
Participants	6	(a) Cohort study **–** give the eligibility criteria, and the sources and methods of selection of participants. Describe methods of follow-upCase-control study **–** give the eligibility criteria, and the sources and methods of case ascertainment and control selection. Give the rationale for the choice of cases and controlsCross-sectional study **–** give the eligibility criteria and the sources and methods of selection of participants	Give information on the criteria and methods for selection of subsets of participants from a larger study, when relevant
		(b) Cohort study **–** for matched studies, give matching criteria and number of exposed and unexposedCase-control study **–** for matched studies, give matching criteria and the number of controls per case	
Variables	7	(a) Clearly define all outcomes, exposures, predictors, potential confounders and effect modifiers. Give diagnostic criteria, if applicable	(b) Clearly define genetic exposures (genetic variants) using a widely-used nomenclature system. Identify variables likely to be associated with population stratification (confounding by ethnic origin)
Data sources/measurement	8*	(a) For each variable of interest, give sources of data and details of methods of assessment (measurement). Describe comparability of assessment methods, if there is more than one group	(b) Describe laboratory methods, including source and storage of DNA, genotyping methods and platforms (including the allele calling algorithm used and its version), error rates and call rates. State the laboratory/centre where genotyping was done. Describe comparability of laboratory methods, if there is more than one group. Specify whether genotypes were assigned using all of the data from the study simultaneously or in smaller batches
Bias	9	(a) Describe any efforts to address potential sources of bias	(b) For quantitative outcome variables, specify if any investigation of potential bias resulting from pharmacotherapy was undertaken. If relevant, describe the nature and magnitude of the potential bias and explain what approach was used to deal with this
Study size	10	Explain how the study size was arrived at	
Quantitative variables	11	Explain how quantitative variables were handled in the analyses. If applicable, describe which groupings were chosen and why	If applicable, describe how effects of treatment were dealt with
Statistical methods	12	(a) Describe all statistical methods, including those used to control for confounding	State software version used and options (or settings) chosen
		(b) Describe any methods used to examine subgroups and interactions	
		(c) Explain how missing data were addressed	
		(d) Cohort study **–** if applicable, explain how loss to follow-up was addressed Case-control study **–** if applicable, explain how matching of cases and controls was addressed Cross-sectional study **–** if applicable, describe analytical methods taking account of sampling strategy	
		(e) Describe any sensitivity analyses	
			(f) State whether Hardy–Weinberg equilibrium was considered and, if so, how
			(g) Describe any methods used for inferring genotypes or haplotypes
			(h) Describe any methods used to assess or address population stratification
			(i) Describe any methods used to address multiple comparisons or to control risk of false positive findings
			(j) Describe any methods used to address and correct for relatedness among subjects
Results			
Participants	13*	(a) Report the numbers of individuals at each stage of the study – e.g., numbers potentially eligible, examined for eligibility, confirmed eligible, included in the study, completing follow-up and analysed	Report numbers of individuals in whom genotyping was attempted and numbers of individuals in whom genotyping was successful
		(b) Give reasons for non-participation at each stage	
		(c) Consider use of a flow diagram	
Descriptive data	14*	(a) Give characteristics of study participants (e.g., demographic, clinical, social) and information on exposures and potential confounders	Consider giving information by genotype
		(b) Indicate the number of participants with missing data for each variable of interest	
		(c) Cohort study **–** summarize follow-up time, e.g. average and total amount	
Outcome data	15*	Cohort study **–** report numbers of outcome events or summary measures over time	Report outcomes (phenotypes) for each genotype category over time
		Case-control study **–** report numbers in each exposure category or summary measures of exposure	Report numbers in each genotype category
		Cross-sectional study **–** report numbers of outcome events or summary measures	Report outcomes (phenotypes) for each genotype category
Main results	16	(a) Give unadjusted estimates and, if applicable, confounder-adjusted estimates and their precision (e.g., 95% confidence intervals). Make clear which confounders were adjusted for and why they were included	
		(b) Report category boundaries when continuous variables were categorized	
		(c) If relevant, consider translating estimates of relative risk into absolute risk for a meaningful time period	
			(d) Report results of any adjustments for multiple comparisons
Other analyses	17	(a) Report other analyses performed – e.g., analyses of subgroups and interactions and sensitivity analyses	
			(b) If numerous genetic exposures (genetic variants) were examined, summarize results from all analyses undertaken
			(c) If detailed results are available elsewhere, state how they can be accessed
Discussion			
Key results	18	Summarize key results with reference to study objectives	
Limitations	19	Discuss limitations of the study, taking into account sources of potential bias or imprecision. Discuss both direction and magnitude of any potential bias	
Interpretation	20	Give a cautious overall interpretation of results considering objectives, limitations, multiplicity of analyses, results from similar studies and other relevant evidence	
Generalizability	21	Discuss the generalizability (external validity) of the study results	
Other information			
Funding	22	Give the source of funding and the role of the funders for the present study and, if applicable, for the original study on which the present article is based	

STREGA, STrengthening the REporting of Genetic Association studies; STROBE, Strengthening the Reporting of Observational Studies in Epidemiology.

* Give information separately for cases and controls in case-control studies and, if applicable, for exposed and unexposed groups in cohort and cross-sectional studies.

**Table 2:** Rationale for inclusion of topics in the STREGA recommendations

Specific issue in genetic association studies	Rationale for inclusion in STREGA	Item(s) in STREGA	Specific suggestions for reporting
Main areas of special interest **(**See also main text)			
Genotyping errors (misclassification of exposure)	Non-differential genotyping errors will usually bias associations towards the null [65,66]. When there are systematic differences in genotyping according to outcome status (differential error), bias in any direction may occur	8(b): Describe laboratory methods, including source and storage of DNA, genotyping methods and platforms (including the allele calling algorithm used, and its version), error rates and call rates. State the laboratory/centre where genotyping was performed. Describe comparability of laboratory methods if there is more than one group. Specify whether genotypes were assigned using all of the data from the study simultaneously or in smaller batches 13(a): Report numbers of individuals in whom genotyping was attempted and numbers of individuals in whom genotyping was successful	Factors affecting the potential extent of misclassification (information bias) of genotype include the types and quality of samples, timing of collection and the method used for genotyping [18,61,136] When high throughput platforms are used, it is important to report not only the platform used but also the allele calling algorithm and its version. Different calling algorithms have different strengths and weaknesses ([130] and supplementary information in [85]). For example, some of the currently used algorithms are notably less accurate in assigning genotypes to single nucleotide polymorphisms with low minor allele frequencies (< 0.10) than to single nucleotide polymorphisms with higher minor allele frequencies [129]. Algorithms are continually being improved. Reporting the allele calling algorithm and its version will help readers to interpret reported results, and it is critical for reproducing the results of the study given the same intermediate output files summarizing intensity of hybridization For some high throughput platforms, the user may choose to assign genotypes using all of the data from the study simultaneously, or in smaller batches, such as by plate ([68,137] and supplementary information [85]). This choice can affect both the overall call rate and the robustness of the calls For case-control studies, whether genotyping was done blind to case-control status should be reported, along with the reason for this decision
Population stratification (confounding by ethnic origin)	When study sub-populations differ both in allele (or genotype) frequencies and disease risks, then confounding will occur, if these sub-populations are unevenly distributed across exposure groups (or between cases and controls)	12(h): Describe any methods used to assess or address population stratification	In view of the debate about the potential implications of population stratification for the validity of genetic association studies, transparent reporting of the methods used, or stating that none was used, to address this potential problem is important for allowing the empirical evidence to accrue Ethnicity information should be presented (see for example Winker [138]), as should genetic markers or other variables likely to be associated with population stratification. Details of case-family control designs should be provided, if they are used As several methods of adjusting for population stratification have been proposed [84], explicit documentation of the methods is needed
Modelling haplotype variation	In designs considered in this article, haplotypes have to be inferred because of lack of available family information. There are diverse methods for inferring haplotypes	12(g): Describe any methods used for inferring genotypes or haplotypes	When discrete ‘windows’ are used to summarize haplotypes, variation in the definition of these may complicate comparisons across studies, as results may be sensitive to choice of windows. Related ‘imputation’ strategies are also in use [85,91,139] It is important to give details on haplotype inference and, when possible, uncertainty. Additional considerations for reporting include the strategy for dealing with rare haplotypes, window size and construction (if used) and choice of software
Hardy–Weinberg equilibrium (HWE)	Departure from Hardy–Weinberg equilibrium may indicate errors or peculiarities in the data [128]. Empirical assessments have found that 20%–69% of genetic associations were reported with some indication about conformity with Hardy–Weinberg equilibrium, and that among some of these, there were limitations or errors in its assessment [128]	12(f): State whether Hardy–Weinberg equilibrium was considered and, if so, how	Any statistical tests or measures should be described, as should any procedure to allow for deviations from Hardy–Weinberg equilibrium in evaluating genetic associations [131]
Replication	Publications that present and synthesize data from several studies in a single report are becoming more common	3: State if the study is the first report of a genetic association, a replication effort, or both	The selected criteria for claiming successful replication should also be explicitly documented
Additional issues			
Selection of participants	Selection bias may occur if (i) genetic associations are investigated in one or more subsets of participants (sub-samples) from a particular study; or (ii) there is differential non-participation in groups being compared; or, (iii) there are differential genotyping call rates in groups being compared	6(a): Give information on the criteria and methods for selection of subsets of participants from a larger study, when relevant 13(a): Report numbers of individuals in whom genotyping was attempted and numbers of individuals in whom genotyping was successful	Inclusion and exclusion criteria, sources and methods of selection of sub-samples should be specified, stating whether these were based on a priori or post-hoc considerations
Rationale for choice of genes and variants investigated	Without an explicit rationale, it is difficult to judge the potential for selective reporting of study results. There is strong empirical evidence from randomised controlled trials that reporting of trial outcomes is frequently incomplete and biased in favour of statistically significant findings [140–142]. Some evidence is also available in pharmacogenetics [143]	7(b): Clearly define genetic exposures (genetic variants) using a widely-used nomenclature system. Identify variables likely to be associated with population stratification (confounding by ethnic origin)	The scientific background and rationale for investigating the genes and variants should be reported For genome-wide association studies, it is important to specify what initial testing platforms were used and how gene variants are selected for further testing in subsequent stages. This may involve statistical considerations (for example, selection of *P-*value threshold), functional or other biological considerations, fine mapping choices, or other approaches that need to be specified Guidelines for human gene nomenclature have been published by the Human Gene Nomenclature Committee [144,145]. Standard reference numbers for nucleotide sequence variations, largely but not only SNPs are provided in dbSNP, the National Center for Biotechnology Information’s database of genetic variation [146]. For variations not listed in dbSNP that can be described relative to a specified version, guidelines have been proposed [147,148]
Treatment effects in studies of quantitative traits	A study of a quantitative variable may be compromised when the trait is subjected to the effects of a treatment, for example, the study of a lipid-related trait for which several individuals are taking lipid-lowering medication. Without appropriate correction, this can lead to bias in estimating the effect and loss of power	9(b): For quantitative outcome variables, specify if any investigation of potential bias resulting from pharmacotherapy was undertaken. If relevant, describe the nature and magnitude of the potential bias, and explain what approach was used to deal with this 11: If applicable, describe how effects of treatment were dealt with	Several methods of adjusting for treatment effects have been proposed [149]. As the approach to deal with treatment effects may have an important impact on both the power of the study and the interpretation of the results, explicit documentation of the selected strategy is needed
Statistical methods	Analysis methods should be transparent and replicable, and genetic association studies are often performed using specialized software	12(a): State software version used and options (or settings) chosen	
Relatedness	The methods of analysis used in family-based studies are different from those used in studies that are based on unrelated cases and controls. Moreover, even in the studies that are based on apparently unrelated cases and controls, some individuals may have some connection and may be (distant) relatives, and this is particularly common in small, isolated populations, for example, Iceland. This may need to be probed with appropriate methods and adjusted for in the analysis of the data	12(j): Describe any methods used to address and correct for relatedness among subjects	For the great majority of studies in which samples are drawn from large, non-isolated populations, relatedness is typically negligible and results would not be altered depending on whether relatedness is taken into account. This may not be the case in isolated populations or those with considerable inbreeding. If investigators have assessed for relatedness, they should state the method used [150–152] and how the results are corrected for identified relatedness
Reporting of descriptive and outcome data	The synthesis of findings across studies depends on the availability of sufficiently detailed data	14(a): Consider giving information by genotype 15: Cohort study – Report outcomes (phenotypes) for each genotype category over time Case-control study – Report numbers in each genotype category Cross-sectional study – Report outcomes (phenotypes) for each genotype category	
Volume of data	The key problem is of possible false-positive results and selective reporting of these. Type I errors are particularly relevant to the conduct of genome-wide association studies. A large search among hundreds of thousands of genetic variants can be expected by chance alone to find thousands of false positive results (odds ratios significantly different from 1.0)	12(i): Describe any methods used to address multiple comparisons or to control risk of false positive findings 16(d): Report results of any adjustments for multiple comparisons 17(b): If numerous genetic exposures (genetic variants) were examined, summarize results from all analyses undertaken 17(c): If detailed results are available elsewhere, state how they can be accessed	Genome-wide association studies collect information on a very large number of genetic variants concomitantly. Initiatives to make the entire database transparent and available online may supply a definitive solution to the problem of selective reporting [7] Availability of raw data may help interested investigators reproduce the published analyses and also pursue additional analyses. A potential drawback of public data availability is that investigators using the data second-hand may not be aware of limitations or other problems that were originally encountered, unless these are also transparently reported. In this regard, collaboration of the data users with the original investigators may be beneficial. Issues of consent and confidentiality [153,154] may also complicate what data can be shared, and how. It would be useful for published reports to specify not only what data can be accessed and where, but also briefly mention the procedure. For articles that have used publicly available data, it would be useful to clarify whether the original investigators were also involved and if so, how The volume of data analysed should also be considered in the interpretation of findings Examples of methods of summarizing results include giving distribution of *P-*values (frequentist statistics), distribution of effect sizes and specifying false discovery rates

STREGA, STrengthening the Reporting of Genetic Association studies; SNP, single nucleotide polymorphism.

### Genotyping errors

Recommendation for reporting of methods [(Table 1), item 8(b)]: Describe laboratory methods, including source and storage of DNA, genotyping methods and platforms (including the allele calling algorithm used, and its version), error rates and call rates. State the laboratory/centre where genotyping was done. Describe comparability of laboratory methods if there is more than one group. Specify whether genotypes were assigned using all of the data from the study simultaneously or in smaller batches.

Recommendation for reporting of results [(Table 1), item 13(a)]: Report numbers of individuals in whom genotyping was attempted and numbers of individuals in whom genotyping was successful.

Genotyping errors can occur as a result of effects of the DNA sequence flanking the marker of interest, poor quality or quantity of the DNA extracted from biological samples, biochemical artefacts, poor equipment precision or equipment failure, or human error in sample handling, conduct of the array or handling the data obtained from the array [61]. A commentary published in 2005 on the possible causes and consequences of genotyping errors observed that an increasing number of researchers were aware of the problem, but that the effects of such errors had largely been neglected [61]. The magnitude of genotyping errors has been reported to vary between 0.5% and 30% [61–64]. In high-throughput centres, an error rate of 0.5% per genotype has been observed for blind duplicates that were run on the same gel [64]. This lower error rate reflects an explicit choice of markers for which genotyping rates have been found to be highly repeatable and the individual polymerase chain reactions of which have been optimized. Non-differential genotyping errors, that is, those that do not differ systematically according to outcome status, will usually bias associations towards the null [65,66], just as for other non-differential errors. The most marked bias occurs when genotyping sensitivity is poor and genotype prevalence is high (> 85%) or, as the corollary, when genotyping specificity is poor and genotype prevalence is low (< 15%) [65]. When measurement of the environmental exposure has substantial error, genotyping errors of the order of 3% can lead to substantial under-estimation of the magnitude of an interaction effect [67]. When there are systematic differences in genotyping according to outcome status (differential error), bias in any direction may occur. Unblinded assessment may lead to differential misclassification. For genome-wide association studies of SNPs, differential misclassification between comparison groups (for example, cases and controls) can occur because of differences in DNA storage, collection or processing protocols, even when the genotyping itself meets the highest possible standards [68]. In this situation, using samples blinded to comparison group to determine the parameters for allele calling could still lead to differential misclassification. To minimize such differential misclassification, it would be necessary to calibrate the software separately for each group. This is one of the reasons for our recommendation to specify whether genotypes were assigned using all of the data from the study simultaneously or in smaller batches.

### Population stratification

Recommendation for reporting of methods [(Table 1), item 12(h)]: Describe any methods used to assess or address population stratification.

Population stratification is the presence within a population of subgroups among which allele (or genotype; or haplotype) frequencies and disease risks differ. When the groups compared in the study differ in their proportions of the population subgroups, an association between the genotype and the disease being investigated may reflect the genotype being an indicator identifying a population subgroup rather than a causal variant. In this situation, population subgroup is a confounder because it is associated with both genotype frequency and disease risk. The potential implications of population stratification for the validity of genetic association studies have been debated [69–83]. Modelling the possible effect of population stratification (when no effort has been made to address it) suggests that the effect is likely to be small in most situations [75,76,78–80]. Meta-analyses of 43 gene-disease associations comprising 697 individual studies showed consistent associations across groups of different ethnic origin [80] and thus provide evidence against a large effect of population stratification, hidden or otherwise. However, as studies of association and interaction typically address moderate or small effects and hence require large sample sizes, a small bias arising from population stratification may be important [81]. Study design (case-family control studies) and statistical methods [84] have been proposed to address population stratification, but so far, few studies have used these suggestions [49]. Most of the early genome-wide association studies used family-based designs or such methods as genomic control and principal components analysis [85,86] to control for stratification. These approaches are particularly appropriate for addressing bias when the identified genetic effects are very small (odds ratio < 1.20), as has been the situation in many recent genome-wide association studies [85,87–105]. In view of the debate on the potential implications of population stratification for the validity of genetic association studies, we recommend transparent reporting of the methods used, or stating that none was used, to address this potential problem. This reporting will enable empirical evidence to accrue about the effects of population stratification and methods to address it.

### Modelling haplotype variation

Recommendation for reporting of methods [(Table 1), item 12(g)]: Describe any methods used for inferring genotypes or haplotypes.

A haplotype is a combination of specific alleles at neighbouring genes that tends to be inherited together. There has been considerable interest in modelling haplotype variation within candidate genes. Typically, the number of haplotypes observed within a gene is much smaller than the theoretical number of all possible haplotypes [106,107]. Motivation for utilizing haplotypes comes, in large part, from the fact that multiple SNPs may ‘tag’ an untyped variant more effectively than a single typed variant. The subset of SNPs used in such an approach is called ‘haplotype tagging’ SNPs. Implicitly, an aim of haplotype tagging is to reduce the number of SNPs that have to be genotyped, while maintaining statistical power to detect an association with the phenotype. Maps of human genetic variation are becoming more complete and large scale genotypic analysis is becoming increasingly feasible. In consequence, it is possible that modelling haplotype variation will become more focused on rare causal variants, because these may not be included in the genotyping platforms.

In most current large-scale genetic association studies, data are collected as unphased multilocus genotypes (that is, which alleles are aligned together on particular segments of chromosome is unknown). It is common in such studies to use statistical methods to estimate haplotypes [108–111] and their accuracy and efficiency have been discussed [112–116]. Some methods attempt to make use of a concept called haplotype ‘blocks’ [117,118], but the results of these methods are sensitive to the specific definitions of the ‘blocks’ [119,120]. Reporting of the methods used to infer individual haplotypes and population haplotype frequencies, along with their associated uncertainties should enhance our understanding of the possible effects of different methods of modelling haplotype variation on study results as well as enabling comparison and syntheses of results from different studies.

Information on common patterns of genetic variation revealed by the International Haplotype Map (HapMap) Project [107] can be applied in the analysis of genome-wide association studies to infer genotypic variation at markers not typed directly in these studies [121,122]. Essentially, these methods perform haplotype-based tests, but make use of information on variation in a set of reference samples (for example, HapMap) to guide the specific tests of association, collapsing a potentially large number of haplotypes into two classes (the allelic variation) at each marker. It is expected that these techniques will increase power in individual studies, and will aid in combining data across studies and even across differing genotyping platforms. If imputation procedures have been used, it is useful to know the method, accuracy thresholds for acceptable imputation, how imputed genotypes were handled or weighted in the analysis and whether any associations based on imputed genotypes were also verified on the basis of direct genotyping at a subsequent stage.

### Hardy–Weinberg equilibrium

Recommendation for reporting of methods [(Table 1), item 12(f)]: State whether Hardy–Weinberg equilibrium was considered and, if so, how.

Hardy–Weinberg equilibrium has become widely accepted as an underlying model in population genetics after Hardy [123] and Weinberg [124] proposed the concept that genotype frequencies at a genetic locus are stable within one generation of random mating; the assumption of HWE is equivalent to the independence of two alleles at a locus. Views differ on whether testing for departure from HWE is a useful method to detect errors or peculiarities in the data set and also the method of testing [125]. In particular, it has been suggested that deviation from HWE may be a sign of genotyping errors [126–128]. Testing for departure from HWE has a role in detecting gross errors of genotyping in large-scale genotyping projects such as identifying SNPs for which the clustering algorithms used to call genotypes have broken down [85,129]. However, the statistical power to detect less important errors of genotyping by testing for departure from HWE is low [130] and, in hypothetical data, the presence of HWE was generally not altered by the introduction of genotyping errors [131]. Furthermore, the assumptions underlying HWE, including random mating, lack of selection according to genotype and absence of mutation or gene flow, are rarely met in human populations [132,133]. In five of 42 gene-disease associations assessed in meta-analyses of almost 600 studies, the results of studies that violated HWE significantly differed from results of studies that conformed to the model [134]. Moreover, the study suggested that exclusion of HWE-violating studies may result in loss of the statistical significance of some postulated gene-disease associations and that adjustment for the magnitude of deviation from the model may also have the same consequence for some other gene-disease associations. Given the differing views about the value of testing for departure from HWE and about the test methods, transparent reporting of whether such testing was performed and, if so, the method used, is important for allowing the empirical evidence to accrue.

For massive-testing platforms, such as genome-wide association studies, it might be expected that many false-positive violations of HWE would occur if a lenient *P-*value threshold were set. There is no consensus on the appropriate *P-*value threshold for HWE-related quality control in this setting. So, we recommend that investigators state which threshold they have used, if any, to exclude specific polymorphisms from further consideration. For SNPs with low minor allele frequencies, substantially more significant results than expected by chance have been observed and the distribution of alleles at these loci has often been found to show departure from HWE.

For genome-wide association studies, another approach that has been used to detect errors or peculiarities in the data set (due to population stratification, genotyping error, HWE deviations or other reasons) has been to construct quantile-quantile (Q/Q) plots whereby observed association statistics or calculated *P-*values for each SNP are ranked in order from the smallest to the largest and plotted against the expected null distribution [129,130]. The shape of the curve can lend insight into whether or not systematic biases are present.

### Replication

Recommendation: State if the study is the first report of a genetic association, a replication effort, or both (Table 1, item 3).

Articles that present and synthesize data from several studies in a single report are becoming more common. In particular, many genome-wide association analyses describe several different study populations, sometimes with different study designs and genotyping platforms and in various stages of discovery and replication [129,130]. When data from several studies are presented in a single original report, each of the constituent studies and the composite results should be fully described. For example, a discussion of sample size and the reason for arriving at that size would include clear differentiation between the initial group (those that were typed with the full set of SNPs) and those that were included in the replication phase only (typed with a reduced set of SNPs) [129,130]. Describing the methods and results in sufficient detail would require substantial space in print, but options for publishing additional information on the study online make this possible.

## Discussion

The choices made for study design, conduct and data analysis potentially influence the magnitude and direction of results of genetic association studies. However, the empirical evidence on these effects is insufficient. Transparency of reporting is thus essential for developing a better evidence base (Table 2). Transparent reporting helps address gaps in empirical evidence [45], such as the effects of incomplete participation and genotyping errors. It will also help assess the impact of currently controversial issues such as population stratification, methods of inferring haplotypes, departure from HWE and multiple testing on effect estimates under different study conditions.

The STREGA Statement proposes a minimum checklist of items for reporting genetic association studies. The statement has several strengths. First, it is based on existing guidance on reporting observational studies (STROBE). Second, it was developed from discussions of an interdisciplinary group that included epidemiologists, geneticists, statisticians, journal editors and graduate students, thus reflecting a broad collaborative approach in terminology accessible to scientists from diverse disciplines. Finally, it explicitly describes the rationale for the decisions (Table 2) and has a clear plan for dissemination and evaluation.

The STREGA recommendations are available at: http://www.strega-statement.org/. We welcome comments, which will be used to refine future versions of the recommendations. We note that little is known about the most effective ways to apply reporting guidelines in practice and that therefore it has been suggested that editors and authors collect, analyse and report their experiences in using such guidelines [135]. We consider that the STREGA recommendations can be used by authors, peer reviewers and editors to improve the reporting of genetic association studies. We invite journals to endorse STREGA, for example by including STREGA and its Web address in their Instructions for Authors and by advising authors and peer reviewers to use the checklist as a guide. It has been suggested that reporting guidelines are most helpful if authors keep the general content of the guideline items in mind as they write their initial drafts, then refer to the details of individual items as they critically appraise what they have written during the revision process [135]. We emphasize that the STREGA reporting guidelines should not be used for screening submitted manuscripts to determine the quality or validity of the study being reported. Adherence to the recommendations may make some manuscripts longer and this may be seen as a drawback in an era of limited space in a print journal. However, the ability to post-information on the Web should alleviate this concern. The place in which supplementary information is presented can be decided by authors and editors of the individual journal.

We hope that the recommendations stimulate transparent and improved reporting of genetic association studies. In turn, better reporting of original studies would facilitate the synthesis of available research results and the further development of study methods in genetic epidemiology with the ultimate goal of improving the understanding of the role of genetic factors in the cause of diseases.

## Publication in other journals

In order to encourage dissemination of the STREGA Statement, this article has also been published by *Annals of Internal Medicine*, *European Journal of Epidemiology*, *Genetic Epidemiology*, *Human Genetics*, *Journal of Clinical Epidemiology* and *PLoS Medicine*.

Please note that M. J. Khoury and M. Gwinn are US Federal Government employees and therefore copyright for these authors is in the public domain.

## Addresses

Canada Research Chair in Human Genome Epidemiology Ottawa, ON, Canada (J. Little); Department of Epidemiology and Community Medicine, University of Ottawa, Ottawa, ON, Canada (J. Little, D. Moher, K. Hutchings, C. Y. Johnson, V. Tait, M. Wiens, N. Birkett); MRC Biostatistics Unit, Cambridge, UK(J. P.T. Higgins); Department of Hygiene and Epidemiology, School of Medicine, University of Ioannina, Ioannina, Greece; and Center for Genetic Epidemiology and Modeling, Tufts University School of Medicine, Boston, MA, USA(J. P. A. Ioannidis); CIHR New Investigator and Canada Research Chair in Genetic Epidemiology, University of Toronto, Dalla Lana School of Public Health, Toronto, ON, Canada (F. Gagnon); Institute of Social and Preventive Medicine, University of Bern, Bern, Switzerland; and German Cochrane Centre, Department of Medical Biometry and Medical Informatics, University Medical Centre, Freiburg, Germany (E. von Elm); National Office of Public Health Genomics, Centers for Disease Control and Prevention, Atlanta, GA, USA (M. J. Khoury, M. Gwinn); former Senior Editor, Public Library of Science, San Francisco, CA, USA (B. Cohen); MRC Centre for Causal Analyses in Translational Epidemiology, Department of Social Medicine, University of Bristol, Bristol, UK (G. Davey-Smith); Canada Research Chair in Health Knowledge Transfer and Uptake, Clinical Epidemiology Program, Ottawa Health Research Institute, Department of Medicine, University of Ottawa, Ottawa, ON, Canada (J. Grimshaw); University of Texas, MD Anderson Cancer Center, Department of Epidemiology, Houston, TX, USA (P. Scheet); Deputy Editor, *American Journal of Human Genetics*, Boston, MA, USA (R. E. Williamson); Department of Epidemiology and Biostatistics, University of Western Canada; and Robarts Clinical Trials, Robarts Research Institute, London, ON, Canada (G. Yong Zou); Editor, *Paediatric and Perinatal Epidemiology,* Bristol, UK (J. Golding); Editor, *European Journal of Epidemiology*, Rotterdam, The Netherlands (C. van Duijn); Cancer Care Ontario, and Prosserman Centre for Health Research at the Samuel Lunenfeld Research Institute, Toronto, ON, Canada (J. McLaughlin); Canada Research Chair in Genetics of Complex Diseases, Hospital for Sick Children (SickKids), Toronto, ON, Canada (A. Paterson); Director, Cardiovascular Research Methods Centre, University of Ottawa Heart Institute, Ottawa, ON, Canada (G. Wells); Genome Quebec & P3G Observatory, McGill University and Genome Quebec Innovation Center, Montreal, QC, Canada (I. Fortier); Dana-Farber Cancer Institute, Boston, MA, USA (M. Freedman); Senior Editor, *Lancet*, New York, New York, USA (M. Zecevic); Editor, *Genetics in Medicine*, Minneapolis, MN, USA (R. King); Canada Research Chair-James McGill Professor, Department of Epidemiology, Biostatistics and Occupational Health Faculty of Medicine, McGill University, Montreal, QC, Canada (C. Infante-Rivard); University of Ottawa Heart Institute, Ottawa, ON, Canada (A. Stewart).

## References

[1] Khoury MJ, Little J, Burke W, Khoury MJ, Little J, Burke W (2004). Human genome epidemiology: scope and strategies. Human Genome Epidemiology: A Scientific Foundation for Using Genetic Information to Improve Health and Prevent Disease.

[2] Genomics, Health and Society Working Group (2004). Genomics, Health and Society. Emerging Issues for Public Policy.

[3] Lin BK, Clyne M, Walsh M, Gomez O, Yu W, Gwinn M (2006). Tracking the epidemiology of human genes in the literature: the HuGE published literature database. Am J Epidemiol.

[4] Yu Y, Yesupriya A, Clyne M, Wulf A, Gwinn M (2008). HuGE Literature Finder.

[5] Lawrence RW, Evans DM, Cardon LR (2005). Prospects and pitfalls in whole genome association studies. Philos Trans R Soc Lond B Biol Sci.

[6] Thomas DC (2006). Are we ready for genome-wide association studies?. Cancer Epidemiol Biomarkers Prev.

[7] Khoury MJ, Little J, Gwinn M, Ioannidis JP (2007). On the synthesis and interpretation of consistent but weak gene-disease associations in the era of genome-wide association studies. Int J Epidemiol.

[8] Little J, Khoury MJ, Bradley L, Clyne M, Gwinn M (2003). The human genome project is complete. How do we develop a handle for the pump?. Am J Epidemiol.

[9] Ioannidis JP, Bernstein J, Boffetta P, Danesh J, Dolan S (2005). A network of investigator networks in human genome epidemiology. Am J Epidemiol.

[10] Ioannidis JP, Gwinn M, Little J, Higgins JP, Bernstein JL (2006). A road map for efficient and reliable human genome epidemiology. Nat Genet.

[11] von Elm E, Egger M (2004). The scandal of poor epidemiological research. BMJ.

[12] Anonymous (1999). Freely associating (editorial). Nat Genet.

[13] Cardon L, Bell J (2001). Association study designs for complex diseases. Nat Rev Genet.

[14] Weiss S (2001). Association studies in asthma genetics. Am J Respir Crit Care Med.

[15] Weiss ST, Silverman EK, Palmer LJ (2001). Case-control association studies in pharmacogenetics. Pharmacogenomics J.

[16] Cooper DN, Nussbaum RL, Krawczak M (2002). Proposed guidelines for papers describing DNA polymorphism-disease associations. Hum Genet.

[17] Hegele R (2002). SNP judgements and freedom of association. Arterioscler Thromb Vasc Biol.

[18] Little J, Bradley L, Bray MS, Clyne M, Dorman J (2002). Reporting, appraising, and integrating data on genotype prevalence and gene-disease associations. Am J Epidemiol.

[19] Romero R, Kuivaniemi H, Tromp G, Olson JM (2002). The design, execution, and interpretation of genetic association studies to decipher complex diseases. Am J Obstet Gynecol.

[20] Colhoun HM, McKeigue PM, Davey Smith G (2003). Problems of reporting genetic associations with complex outcomes. Lancet.

[21] van Duijn CM, Porta M (2003). Good prospects for genetic and molecular epidemiologic studies in the European Journal of Epidemiology. Eur J Epidemiol.

[22] Crossman D, Watkins H (2004). Jesting pilate, genetic case-control association studies, and heart. Heart.

[23] Huizinga TW, Pisetsky DS, Kimberly RP (2004). Associations, populations, and the truth: recommendations for genetic association studies in arthritis & rheumatism. Arthritis Rheum.

[24] Little J, Khoury MJ, Little J, Burke W (2004). Reporting and review of human genome epidemiology studies. Human Genome Epidemiology: A Scientific Foundation for Using Genetic Information to Improve Health and Prevent Disease.

[25] Rebbeck TR, Martinez ME, Sellers TA, Shields PG, Wild CP (2004). Genetic variation and cancer: improving the environment for publication of association studies. Cancer Epidemiol Biomarkers Prev.

[26] Tan N, Mulley J, Berkovic S (2004). Association studies in epilepsy: “The truth is out there”. Epilepsia.

[27] Anonymous (2005). Framework for a fully powered risk engine. Nat Genet.

[28] Ehm MG, Nelson MR, Spurr NK (2005). Guidelines for conducting and reporting whole genome/large-scale association studies. Hum Mol Genet.

[29] Freimer NB, Sabatti C (2005). Guidelines for association studies in human molecular genetics. Hum Mol Genet.

[30] Hattersley AT, McCarthy MI (2005). What makes a good genetic association study?. Lancet.

[31] Manly K (2005). Reliability of statistical associations between genes and disease. Immunogenetics.

[32] Shen H, Liu Y, Liu P, Recker R, Deng H (2005). Nonreplication in genetic studies of complex diseases – Lessons learned from studies of osteoporosis and tentative remedies. J Bone Miner Res.

[33] Vitali S, Randolph A (2005). Assessing the quality of case-control association studies on the genetic basis of sepsis. Pediatr Crit Care Med.

[34] Wedzicha JA, Hall IP (2005). Publishing genetic association studies in thorax. Thorax.

[35] Hall IP, Blakey JD (2005). Genetic association studies in thorax. Thorax.

[36] DeLisi LE, Faraone SV (2006). When is a “positive” association truly a “positive” in psychiatric genetics? A commentary based on issues debated at the world congress of psychiatric genetics, Boston, October 12-18, 2005. Am J Med Genet B Neuropsychiatr Genet.

[37] Saito YA, Talley NJ, de Andrade M, Petersen GM (2006). Case-control genetic association studies in gastrointestinal disease: review and recommendations. Am J Gastroenterol.

[38] Uhlig K, Menon V, Schmid CH (2007). Recommendations for reporting of clinical research studies. Am J Kidney Dis.

[39] Chanock SJ, Manolio T, Boehnke M, Boerwinkle E, NCI-NHGRI Working Group on Replication in Association Studies (2007). Replicating genotype-phenotype associations. Nature.

[40] Begg CB (2005). Reflections on publication criteria for genetic association studies. Cancer Epidemiol Biomarkers Prev.

[41] Byrnes G, Gurrin L, Dowty J, Hopper JL (2005). Publication policy or publication bias?. Cancer Epidemiol Biomarkers Prev.

[42] Pharoah PD, Dunning AM, Ponder BA, Easton DF (2005). The reliable identification of disease-gene associations. Cancer Epidemiol Biomarkers Prev.

[43] Wacholder S (2005). Publication environment and broad investigation of the genome. Cancer Epidemiol Biomarkers Prev.

[44] Whittemore AS (2005). Genetic association studies: time for a new paradigm?. Cancer Epidemiol Biomarkers Prev.

[45] Bogardus ST, Concato J, Feinstein AR (1999). Clinical epidemiological quality in molecular genetic research. The need for methodological standards. JAMA.

[46] Peters DL, Barber RC, Flood EM, Garner HR, O’Keefe GE (2003). Methodologic quality and genotyping reproducibility in studies of tumor necrosis factor -308 G-->A. A single nucleotide polymorphism and bacterial sepsis: implications for studies of complex traits. Crit Care Med.

[47] Clark MF, Baudouin SV (2006). A systematic review of the quality of genetic association studies in human sepsis. Intensive Care Med.

[48] Lee W, Bindman J, Ford T, Glozier N, Moran P (2007). Bias in psychiatric case-control studies: literature survey. Br J Psychiatry.

[49] Yesupriya A, Evangelou E, Kavvoura FK, Patsopoulos NA, Clyne M (2008). Reporting of human genome epidemiology (HuGE) association studies: an empirical assessment. BMC Med Res Methodol.

[50] Reid MC, Lachs MS, Feinstein AR (1995). Use of methodological standards in diagnostic test research. Getting better but still not good. JAMA.

[51] Brazma A, Hingamp P, Quackenbush J, Sherlock G, Spellman P (2001). Minimum information about a microarray experiment (MIAME) – Toward standards for microarray data. Nat Genet.

[52] Pocock SJ, Collier TJ, Dandreo KJ, de Stavola BL, Goldman MB (2004). Issues in the reporting of epidemiological studies: a survey of recent practice. BMJ.

[53] Altman D, Moher D (2005). Developing guidelines for reporting healthcare research: scientific rationale and procedures. Med Clin (Barc).

[54] Gluud LL (2006). Bias in clinical intervention research. Am J Epidemiol.

[55] von Elm E, Altman DG, Egger M, Pocock SJ, Gotzsche PC (2007). The strengthening the reporting of observational studies in epidemiology (STROBE) statement: guidelines for reporting observational studies. PLoS Med.

[56] Vandenbroucke JP, von Elm E, Altman DG, Gotzsche PC, Mulrow CD (2007). Strengthening the reporting of observational studies in epidemiology (STROBE): explanation and elaboration. Ann Intern Med.

[57] Little J, Higgins JPT (2006). The HuGENet™ HuGE Review Handbook, version 1.0.

[58] Higgins JP, Little J, Ioannidis JP, Bray MS, Manolio TA (2007). Turning the pump handle: evolving methods for integrating the evidence on gene-disease association. Am J Epidemiol.

[59] Altman DG, Schulz KF, Moher D, Egger M, Davidoff F (2001). The revised CONSORT statement for reporting randomized trials: explanation and elaboration. Ann Intern Med.

[60] Moher D, Schultz KF, Altman D (2001). The CONSORT statement: revised recommendations for improving the quality of reports of parallel-group randomized trials. JAMA.

[61] Pompanon F, Bonin A, Bellemain E, Taberlet P (2005). Genotyping errors: causes, consequences and solutions. Nat Rev Genet.

[62] Akey JM, Zhang K, Xiong M, Doris P, Jin L (2001). The effect that genotyping errors have on the robustness of common linkage-disequilibrium measures. Am J Hum Genet.

[63] Dequeker E, Ramsden S, Grody WW, Stenzel TT, Barton DE (2001). Quality control in molecular genetic testing. Nat Rev Genet.

[64] Mitchell AA, Cutler DJ, Chakravarti A (2003). Undetected genotyping errors cause apparent overtransmission of common alleles in the transmission/disequilibrium test. Am J Hum Genet.

[65] Rothman N, Stewart WF, Caporaso NE, Hayes RB (1993). Misclassification of genetic susceptibility biomarkers: implications for case-control studies and cross-population comparisons. Cancer Epidemiol Biomarkers Prev.

[66] Garcia-Closas M, Wacholder S, Caporaso N, Rothman N, Khoury MJ, Little J, Burke W (2004). Inference issues in cohort and case-control studies of genetic effects and gene-environment interactions. Human Genome Epidemiology: A Scientific Foundation for Using Genetic Information to Improve Health and Prevent Disease.

[67] Wong MY, Day NE, Luan JA, Wareham NJ (2004). Estimation of magnitude in gene-environment interactions in the presence of measurement error. Stat Med.

[68] Clayton DG, Walker NM, Smyth DJ, Pask R, Cooper JD (2005). Population structure, differential bias and genomic control in a large-scale, case-control association study. Nat Genet.

[69] Knowler WC, Williams RC, Pettitt DJ, Steinberg AG (1988). Gm3;5,13,14 and type 2 diabetes mellitus: an association in American Indians with genetic admixture. Am J Human Genet.

[70] Gelernter J, Goldman D, Risch N (1993). The A1 allele at the D2 dopamine receptor gene and alcoholism: a reappraisal. JAMA.

[71] Kittles RA, Chen W, Panguluri RK, Ahaghotu C, Jackson A (2002). CYP3A4-V and prostate cancer in African Americans: causal or confounding association because of population stratification?. Hum Genet.

[72] Thomas DC, Witte JS (2002). Point: population stratification: a problem for case control studies of candidate-gene associations?. Cancer Epidemiol Biomarkers Prev.

[73] Wacholder S, Chatterjee N, Hartge P (2002). Joint effects of genes and environment distorted by selection biases: implications for hospital-based case-control studies. Cancer Epidemiol Biomarkers Prev.

[74] Cardon LR, Palmer LJ (2003). Population stratification and spurious allelic association. Lancet.

[75] Wacholder S, Rothman N, Caporaso N (2000). Population stratification in epidemiologic studies of common genetic variants and cancer: quantification of bias. J Natl Cancer Inst.

[76] Ardlie KG, Lunetta KL, Seielstad M (2002). Testing for population subdivision and association in four case-control studies. Am J Human Genet.

[77] Edland SD, Slager S, Farrer M (2004). Genetic association studies in Alzheimer’s disease research: challenges and opportunities. Stat Med.

[78] Millikan RC (2001). Re: population stratification in epidemiologic studies of common genetic variants and cancer: quantification of bias. J Natl Cancer Inst.

[79] Wang Y, Localio R, Rebbeck TR (2004). Evaluating bias due to population stratification in case-control association studies of admixed populations. Genet Epidemiol.

[80] Ioannidis JP, Ntzani EE, Trikalinos TA (2004). ‘Racial’ differences in genetic effects for complex diseases. Nat Genet.

[81] Marchini J, Cardon LR, Phillips MS, Donnelly P (2004). The effects of human population structure on large genetic association studies. Nat Genet.

[82] Freedman ML, Reich D, Penney KL, McDonald GJ, Mignault AA (2004). Assessing the impact of population stratification on genetic association studies. Nat Genet.

[83] Khlat M, Cazes MH, Genin E, Guiguet M (2004). Robustness of case-control studies of genetic factors to population stratification: magnitude of bias and type I error. Cancer Epidemiol Biomarkers Prev.

[84] Balding DJ (2006). A tutorial on statistical methods for population association studies. Nat Rev Genet.

[85] Wellcome Trust Case Control Consortium (2007). Genome-wide association study of 14,000 cases of seven common diseases and 3,000 shared controls. Nature.

[86] Ioannidis JP (2007). Non-replication and inconsistency in the genome-wide association setting. Hum Hered.

[87] Parkes M, Barrett JC, Prescott NJ, Tremelling M, Anderson CA (2007). Sequence variants in the autophagy gene IRGM and multiple other replicating loci contribute to Crohn’s disease susceptibility. Nat Genet.

[88] Todd JA, Walker NM, Cooper JD, Smyth DJ, Downes K (2007). Robust associations of four new chromosome regions from genome-wide analyses of type 1 diabetes. Nat Genet.

[89] Zeggini E, Weedon MN, Lindgren CM, Frayling TM, Elliott KS (2007). Replication of genome-wide association signals in UK samples reveals risk loci for type 2 diabetes. Science.

[90] Saxena R, Voight BF, Lyssenko V, Burtt NP, Diabetes Genetics Initiative of Broad Institute of Harvard and MIT, Lund University, and Novartis Institutes of BioMedical Research (2007). Genome-wide association analysis identifies loci for type 2 diabetes and triglyceride levels. Science.

[91] Scott LJ, Mohlke KL, Bonnycastle LL, Willer CJ, Li Y (2007). A genome-wide association study of type 2 diabetes in Finns detects multiple susceptibility variants. Science.

[92] Helgadottir A, Thorleifsson G, Manolescu A, Gretarsdottir S, Blondal T (2007). A common variant on chromosome 9p21 affects the risk of myocardial infarction. Science.

[93] McPherson R, Pertsemlidis A, Kavaslar N, Stewart A, Roberts R (2007). A common allele on chromosome 9 associated with coronary heart disease. Science.

[94] Easton DF, Pooley KA, Dunning AM, Pharoah PD, Thompson D (2007). Genome-wide association study identifies novel breast cancer susceptibility loci. Nature.

[95] Hunter DJ, Kraft P, Jacobs KB, Cox DG, Yeager M (2007). A genome-wide association study identifies alleles in FGFR2 associated with risk of sporadic postmenopausal breast cancer. Nat Genet.

[96] Stacey SN, Manolescu A, Sulem P, Rafnar T, Gudmundsson J (2007). Common variants on chromosomes 2q35 and 16q12 confer susceptibility to estrogen receptor-positive breast cancer. Nat Genet.

[97] Gudmundsson J, Sulem P, Steinthorsdottir V, Bergthorsson JT, Thorleifsson G (2007). Two variants on chromosome 17 confer prostate cancer risk, and the one in TCF2 protects against type 2 diabetes. Nat Genet.

[98] Haiman CA, Patterson N, Freedman ML, Myers SR, Pike MC (2007). Multiple regions within 8q24 independently affect risk for prostate cancer. Nat Genet.

[99] Yeager M, Orr N, Hayes RB, Jacobs KB, Kraft P (2007). Genome-wide association study of prostate cancer identifies a second risk locus at 8q24. Nat Genet.

[100] Zanke BW, Greenwood CM, Rangrej J, Kustra R, Tenesa A (2007). Genome-wide association scan identifies a colorectal cancer susceptibility locus on chromosome 8q24. Nat Genet.

[101] Tomlinson I, Webb E, Carvajal-Carmona L, Broderick P, Kemp Z (2007). A genome-wide association scan of tag SNPs identifies a susceptibility variant for colorectal cancer at 8q24.21. Nat Genet.

[102] Haiman CA, Le Marchand L, Yamamoto J, Stram DO, Sheng X (2007). A common genetic risk factor for colorectal and prostate cancer. Nat Genet.

[103] Rioux JD, Xavier RJ, Taylor KD, Silverberg MS, Goyette P (2007). Genome-wide association study identifies new susceptibility loci for Crohn disease and implicates autophagy in disease pathogenesis. Nat Genet.

[104] Libioulle C, Louis E, Hansoul S, Sandor C, Farnir F (2007). Novel Crohn disease locus identified by genome-wide association maps to a gene desert on 5p13.1 and modulates expression of PTGER4. PLoS Genet.

[105] Duerr RH, Taylor KD, Brant SR, Rioux JD, Silverberg MS (2006). A genome-wide association study identifies IL23R as an inflammatory bowel disease gene. Science.

[106] Zhao LP, Li SS, Khalid N (2003). A method for the assessment of disease associations with single-nucleotide polymorphism haplotypes and environmental variables in case-control studies. Am J Hum Genet.

[107] Frazer KA, Ballinger DG, Cox DR, Hinds DA, International HapMap Consortium (2007). A second generation human haplotype map of over 3.1 million SNPs. Nature.

[108] Stephens M, Smith NJ, Donnelly P (2001). A new statistical method for haplotype reconstruction from population data. Am J Hum Genet.

[109] Qin ZS, Niu T, Liu JS (2002). Partition-ligation-expectation-maximization algorithm for haplotype inference with single-nucleotide polymorphisms. Am J Hum Genet.

[110] Scheet P, Stephens M (2006). A fast and flexible statistical model for large-scale population genotype data: applications to inferring missing genotypes and haplotypic phase. Am J Hum Genet.

[111] Browning SR (2008). Missing data imputation and haplotype phase inference for genome-wide association studies. Hum Genet.

[112] Huang Q, Fu YX, Boerwinkle E (2003). Comparison of strategies for selecting single nucleotide polymorphisms for case/control association studies. Hum Genet.

[113] Kamatani N, Sekine A, Kitamoto T, Iida A, Saito S (2004). Large-scale single-nucleotide polymorphism (SNP) and haplotype analyses, using dense SNP maps, of 199 drug-related genes in 752 subjects: The analysis of the association between uncommon SNPs within haplotype blocks and the haplotypes constructed with haplotype-tagging SNPs. Am J Hum Genet.

[114] Zhang W, Collins A, Morton NE (2004). Does haplotype diversity predict power for association mapping of disease susceptibility?. Hum Genet.

[115] Carlson CS, Eberle MA, Rieder MJ, Yi Q, Kruglyak L (2004). Selecting a maximally informative set of single-nucleotide polymorphisms for association analysis using linkage disequilibrium. Am J Hum Genet.

[116] van Hylckama Vlieg A, Sandkuijl LA, Rosendaal FR, Bertina RM, Vos HL (2004). Candidate gene approach in association studies: would the factor V leiden mutation have been found by this approach?. Eur J Hum Genet.

[117] Greenspan G, Geiger D (2004). Model-based inference of haplotype block variation. J Comput Biol.

[118] Kimmel G, Shamir R (2005). GERBIL: genotype resolution and block identification using likelihood. Proc Natl Acad Sci USA.

[119] Cardon LR, Abecasis GR (2003). Using haplotype blocks to map human complex triat loci. Trends Genet.

[120] Ke X, Hunt S, Tapper W, Lawrence R, Stavrides G (2004). The impact of SNP density on fine-scale patterns of linkage disequilibrium. Hum Mol Genet.

[121] Servin B, Stephens M (2007). Imputation-based analysis of association studies: candidate regions and quantitative traits. PLoS Genet.

[122] Marchini J, Howie B, Myers S, McVean G, Donnelly P (2007). A new multipoint method for genome-wide association studies by imputation of genotypes. Nat Genet.

[123] Hardy GH (1908). Mendelian proportions in a mixed population. Science.

[124] Weinberg W (1908). Über den nachweis der vererbung beim menschen. Jahrhefte Des Vereines Für Vaterländische Naturkunde in Württemberg.

[125] Minelli C, Thompson JR, Abrams KR, Thakkinstian A, Attia J (2008). How should we use information about HWE in the meta-analyses of genetic association studies?. Int J Epidemiol.

[126] Xu J, Turner A, Little J, Bleecker ER, Meyers DA (2002). Positive results in association studies are associated with departure from Hardy-Weinberg equilibrium: hint for genotyping error?. Hum Genet.

[127] Hosking L, Lumsden S, Lewis K, Yeo A, McCarthy L (2004). Detection of genotyping errors by Hardy–Weinberg equilibrium testing. Eur J Hum Genet.

[128] Salanti G, Amountza G, Ntzani EE, Ioannidis JP (2005). Hardy–Weinberg equilibrium in genetic association studies: an empirical evaluation of reporting, deviations, and power. Eur J Hum Genet.

[129] Pearson TA, Manolio TA (2008). How to interpret a genome-wide association study. JAMA.

[130] McCarthy MI, Abecasis GR, Cardon LR, Goldstein DB, Little J (2008). Genome-wide association studies for complex traits: consensus, uncertainty and challenges. Nat Rev Genet.

[131] Zou GY, Donner A (2006). The merits of testing Hardy–Weinberg equilibrium in the analysis of unmatched case-control data: A cautionary note. Ann Hum Genet.

[132] Shoemaker J, Painter I, Weir BS (1998). A Bayesian characterization of Hardy–Weinberg disequilibrium. Genetics.

[133] Ayres KL, Balding DJ (1998). Measuring departures from Hardy–Weinberg: a Markov chain Monte Carlo method for estimating the inbreeding coefficient. Heredity.

[134] Trikalinos TA, Salanti G, Khoury MJ, Ioannidis JP (2006). Impact of violations and deviations in Hardy–Weinberg equilibrium on postulated gene-disease associations. Am J Epidemiol.

[135] Davidoff F, Batalden P, Stevens D, Ogrinc G, Mooney S (2008). Publication guidelines for improvement studies in health care: evolution of the SQUIRE project. Ann Intern Med.

[136] Steinberg K, Gallagher M, Khoury MJ, Little J, Burke W (2004). Assessing genotypes in human genome epidemiology studies. Human Genome epidemiology: A Scientific Foundation for Using Genetic Information to Improve Health and Prevent Disease.

[137] Plagnol V, Cooper JD, Todd JA, Clayton DG (2007). A method to address differential bias in genotyping in large-scale association studies. PLoS Genet.

[138] Winker MA (2006). Race and ethnicity in medical research: requirements meet reality. J Law Med Ethics.

[139] Scuteri A, Sanna S, Chen WM, Uda M, Albai G (2007). Genome-wide association scan shows genetic variants in the FTO gene are associated with obesity-related traits. PLoS Genet.

[140] Chan AW, Hrobjartsson A, Haahr MT, Gotzsche PC, Altman DG (2004). Empirical evidence for selective reporting of outcomes in randomized trials: comparison of protocols to published articles. JAMA.

[141] Chan AW, Krleza-Jeric K, Schmid I, Altman DG (2004). Outcome reporting bias in randomized trials funded by the Canadian institutes of health research. CMAJ.

[142] Chan AW, Altman DG (2005). Identifying outcome reporting bias in randomised trials on PubMed: review of publications and survey of authors. BMJ.

[143] Contopoulos-Ioannidis DG, Alexiou GA, Gouvias TC, Ioannidis JP (2006). An empirical evaluation of multifarious outcomes in pharmacogenetics: beta-2 adrenoceptor gene polymorphisms in asthma treatment. Pharmacogenet Genomics.

[144] Wain HM, Bruford EA, Lovering RC, Lush MJ, Wright MW (2002). Guidelines for human gene nomenclature. Genomics.

[145] Wain HM, Lush M, Ducluzeau F, Povey S (2002). Genew: the human gene nomenclature database. Nucleic Acids Res.

[146] Sherry ST, Ward MH, Kholodov M, Baker J, Phan L (2001). dbSNP: the NCBI database of genetic variation. Nucleic Acids Res.

[147] Antonarakis SE (1998). Recommendations for a nomenclature system for human gene mutations. Nomenclature working group. Hum Mutat.

[148] den Dunnen JT, Antonarakis SE (2000). Mutation nomenclature extensions and suggestions to describe complex mutations: a discussion. Hum Mutat.

[149] Tobin MD, Sheehan NA, Scurrah KJ, Burton PR (2005). Adjusting for treatment effects in studies of quantitative traits: antihypertensive therapy and systolic blood pressure. Stat Med.

[150] Lynch M, Ritland K (1999). Estimation of pairwise relatedness with molecular markers. Genetics.

[151] Slager SL, Schaid DJ (2001). Evaluation of candidate genes in case-control studies: a statistical method to account for related subjects. Am J Hum Genet.

[152] Voight BF, Pritchard JK (2005). Confounding from cryptic relatedness in case-control association studies. PLoS Genet.

[153] Homer N, Szelinger S, Redman M, Duggan D, Tembe W (2008). Resolving individuals contributing trace amounts of DNA to highly complex mixtures using high-density SNP genotyping microarrays. PLoS Genet.

[154] Zerhouni EA, Nabel EG (2008). Protecting aggregate genomic data. Science.

